# Influence of Windowing and Evaluation of Metal Artifact Reduction Algorithm on Five Different Restorative Materials by Using Different Cone Beam Computed Tomography (CBCT) Scanners: A CBCT Study

**DOI:** 10.7759/cureus.41742

**Published:** 2023-07-11

**Authors:** Ajay Sutare, Ajay Parihar, Prashanthi Reddy, Renu Singh, Varsha AC

**Affiliations:** 1 Department of Oral Medicine and Radiology, Government College of Dentistry Indore, Indore, IND

**Keywords:** restorative dental materials, metallic artifact, cone-beam conventional tomography (cbct), artifact reduction, algorithm

## Abstract

Objectives: To assess the influence of windowing, and to evaluate, and compare the effect of the metal artifact reduction (MAR) and non-metal artifact reduction (non-MAR) algorithms on different high-density restorative dental materials using different cone beam computed tomography (CBCT) devices.

Material and method: Height and diameter of all cylindrical shape metals including amalgam, cobalt-chromium, composite, gutta-percha, and titanium were measured using a digital caliper device. Polymethylmethacrylate block and arch phantom with a cylindrical-shaped perforation containing five different metals were submitted to tomographic acquisition with six different cone beam computed tomographic devices in small fields of view with their MAR enabled and disabled. Windowing was done using ITK-SNAP software (3.8.2) which was used as a contrast medial tool for window level and window width. The data was analyzed for probability distribution using the Kolmogorov-Smirnov test, where a p-value of <0.05 indicated that the data were not normally distributed. The comparison of length and width was done using the Wilcoxon sign rank test. Comparison of categorical variables was done using the Chi-square test where a p-value of <0.05 was considered statistically significant.

Results: Length and width of all these metals measured using MAR and non-MAR CBCT were found to be statistically non-significant (p-value of >0.05). MAR algorithm significantly reduces metals artifact produced by high-density restorative materials (p-value of <0.05).

Conclusion: Amalgam and cobalt-chromium produced more artifacts while composite and gutta-percha did not produce enough artifacts to be reduced by the MAR algorithm. Large window width and high window level would be beneficial to reduce the metal artifact.

## Introduction

Cone beam computed tomography (CBCT) is the most advanced radiographic imaging technology that allows significant, accurate, three-dimensional (3D), and high-resolution images with a low radiation dose for imaging of hard tissue structures [1].

Artifacts are one of the main disadvantages, which may reduce the CBCT image quality and negatively impact the diagnosis process as caused by high-density restorative dental materials e. g. a dental implant, gutta-percha, orthodontic brackets, and metals alloy in the oral cavity. There are various artifacts associated with CBCT, namely x-ray-related, patient-related, scanner-related, and noise image artifacts [2]. To overcome this problem a post-processing tool, the metal artifact reduction algorithm (MAR), has been developed for the purpose of reducing the metal artifact, beam hardening effect, and thereby increasing the image quality [3].

For the visualization of soft and hard tissues in the CBCT images, a post-processing tool, windowing, can be used in their visualization to adjust the grey tone of CBCT images. Windowing is the change in the gray value or gray tone for soft tissue and hard tissue, window width is the large range of pixels of gray value and window level is the center of that pixel range value. The windowing and segmentation can be done by using ITK-SNAP 3.8.2 software (www.itksnap.org) [4].

## Materials and methods

The six different restorative materials, one cold cure resin block, and rubber mold impressions material were made in the laboratory and all the metal scans were done from six different CBCT centers in Madhya Pradesh, India.

Sample preparation

**Figure 1:**
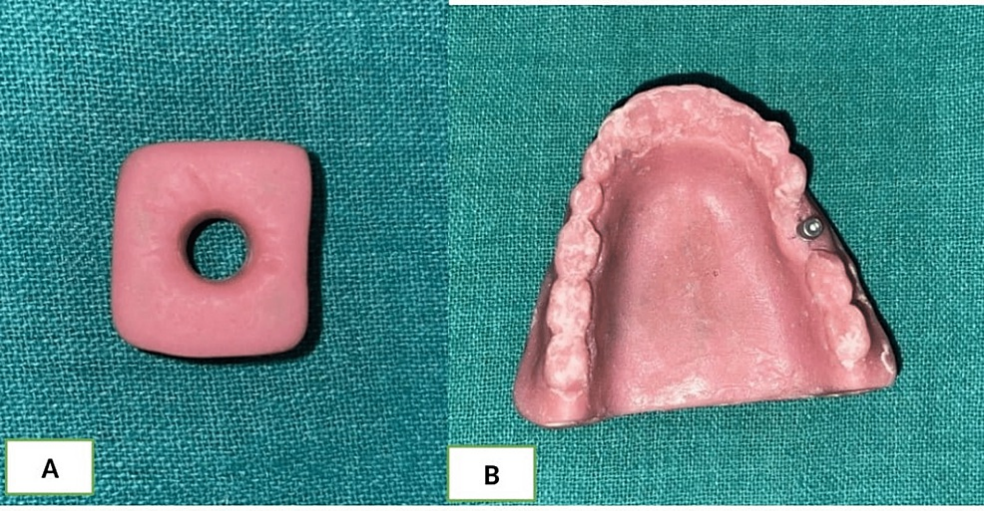
A: The image of polymethylmethacrylate (PMMA) square block with single perforation for cylinder shape metals. B: The image of the PMMA maxillary arch phantom with a titanium implant.

Polymethylmethacrylate (PMMA) (DPI RR Cold Cure; Dental Products of India, Mumbai, India) square block with a cylindrical-shaped perforation in the center along with an arch phantom with single perforation for titanium screw mimicking an upper dental arch was used in this study (Figure 1A, 1B).

**Figure 2:**
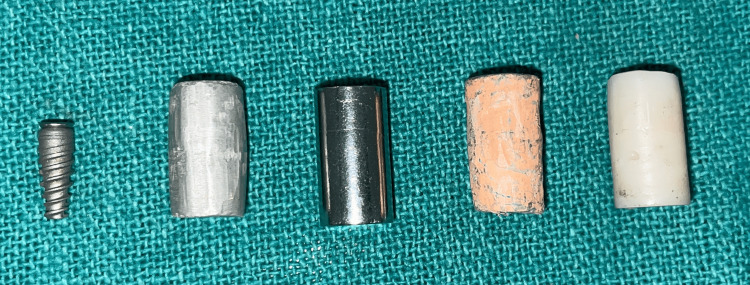
Images of five different cylindrical shape restorative materials, namely titanium, amalgam, cobalt chromium, gutta-percha, and composite.

High-density dental materials were studied including dental amalgam (AM) alloy (Dental Products of India), cobalt-chromium (CO-CR) alloy (Vera PDN; Aalba Dent, Fairfield, CA, USA), composite (CM), gutta-percha (GP) (Sure Dent Corporation, Gyeonggi-do, Korea), and titanium (TI) (Actif Dental Implant Dia 3.7 X 10mm; Artizahn Dental Studio, Canandaigua, NY, USA) (Figure 2).

**Figure 3:**
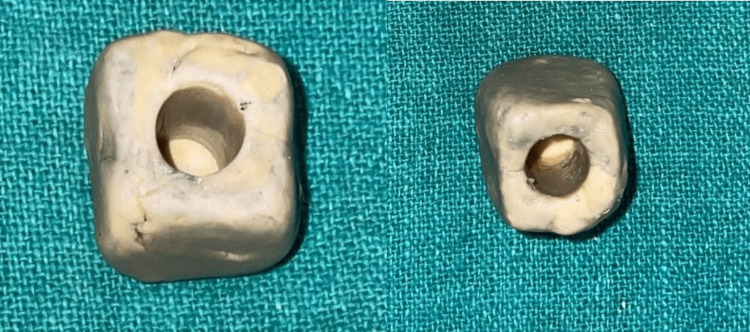
Image of rubber-based impression material mold.

Dental amalgam alloy and gutta-percha were made by condensation process using rubber mold impression material (vinyl polysiloxane impression material KIT light body putty; FLEXCEED Co., Naka, Japan), the composite was made by using light curing method and titanium and cobalt-chromium were ready-made, bought from the local dental depot (Figure 3).

**Figure 4:**
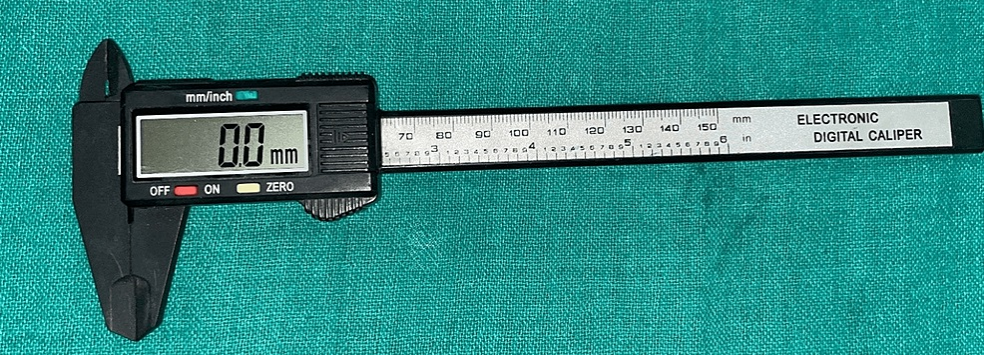
Image of an electronic digital caliper device for measurement of length and width.

The height and diameter of all these metals were measured using a digital caliper device, and repeated measurements were done to ensure high accuracy (Table 1, Figure 4).

**Table 1:** Physical dimensions of each cylinder considering all the dental materials.

Metals	Amalgam	Cobalt-chromium	Composite	Gutta-percha	Titanium
Length	15.0mm	15.0mm	15.0mm	15.5mm	10.6mm
Width	8.5mm	8.1mm	8.1mm	8.5mm	3.7mm

Image acquisition

All of the cylindrical-shaped metals were placed one by one in the PMMA block, and the titanium screw was placed in the PMMA arch phantom to simulate the clinical position of an implant.

CBCT scans were acquired with CS9600 (Carestream Dental, Atlanta, GA, USA) CS8100 (Carestream Dental), ACTEON Trium (de Götzen S.r.l.-ACTEON Group, Olgiate Olona, Italy), CS9300 (Carestream Dental), DENTIUM Rainbow (Dentium, Seoul, South Korea), NewTom (NewTom Cefla S.C., Via Selice, Italy) units.

Exposure parameters were considered for all these CBCT units set by manufacturers using a small field of view (FOV) (Table 2).

**Table 2:** Manufacturer's cone beam computed tomography (CBCT) exposure parameters according to small field of view (FOV).

CBCT MACHINE	VOXEL SPACING	KVP	Voxel Size	X-Ray Tube Current	TIME	DOSE
CS9600	150µmX150µmx150µm	91KV	0.15mm	8mA	10sec	175Gy.cm^2^
CS8100	150µmx150µmX150µm	90KV	0.15mm	5mA	15sec	665mGy.cm^2^
ACTEON Trium	100µmx100µmx100µm	90KV	0.1mm	8mA	10sec	775Gy.cm^2^
CS9300	90µmX90µmx90µm	84KV	0.09mm	8mA	20.00sec	956mGy.cm^2^
DENTIUM Rainbow	190µmX190µMx190µm	94KV	0.190000mm	8mA	10sec	1042Gy.cm
NewTom	150µmX150µMx150µm	90KV	0.150mm	8mA	8sec	6.53mGy

The MAR application was on and off during scanning. All these materials were scanned in the small fields of view using six CBCT units exported in the Digital Imaging and Communications in Medicine (DICOM) format. Windowing was done using ITK-SNAP software (3.8.2) which was used as a contrast medial tool for window level and window width. We kept a standard value of 1000 for window level and 3000 for window width. After this, we compared the manufacturer’s automatic value of all metals and the standard value of all ITK-SNAP software images.

CBCT data collection

**Figure 5:**
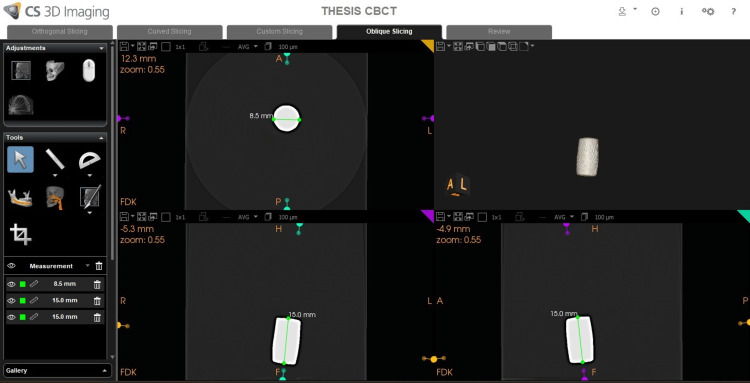
Image of the arrow showing how to use Carestream software for measurement of length and width of metals.

**Figure 6:**
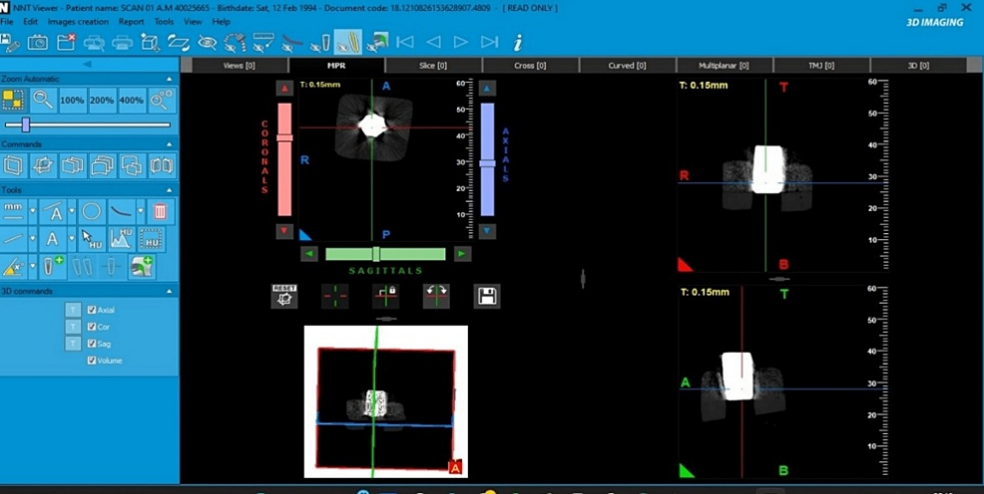
Image of the dark arrow, and a peach color arrow showing using of NewTom NNTVIEWER software in multiplanar reconstructions for measurement of length and width.

All the images of the CBCT scan were uploaded as DICOM files in care stream software. A measurement tool was used to obtain the length and width in all three sections, keeping the middle of each metal in vertical and horizontal directions as standard (Figure 5, 6).

MAR algorithm tool was kept on and off for all metals using three machines including CS9600, CS8100, and ACTEON, and as the remaining three machines did not have MAR algorithm thus, we consider it as non-MAR which includes CS9300, DENTIUM, and NewTom.

We have made ranking orders for an artifact in all the sections, which was evaluated by two oral and maxillofacial radiologists for intra-examiner reliability. A rank was allotted according to the artifacts observed such as 0 was assigned to no artifact, 1 to very less artifact, 2 to less artifact, 3 to more artifacts surrounding metal, and 4 to more artifacts causing distortion of images (Table 3).

**Table 3:** Ranking order of the artifacts.

Criteria	score
No artifact	0
Very less artifact	1
Less artifact	2
More artifact surrounding	3
More artifact image distortion	4

**Figure 7:**
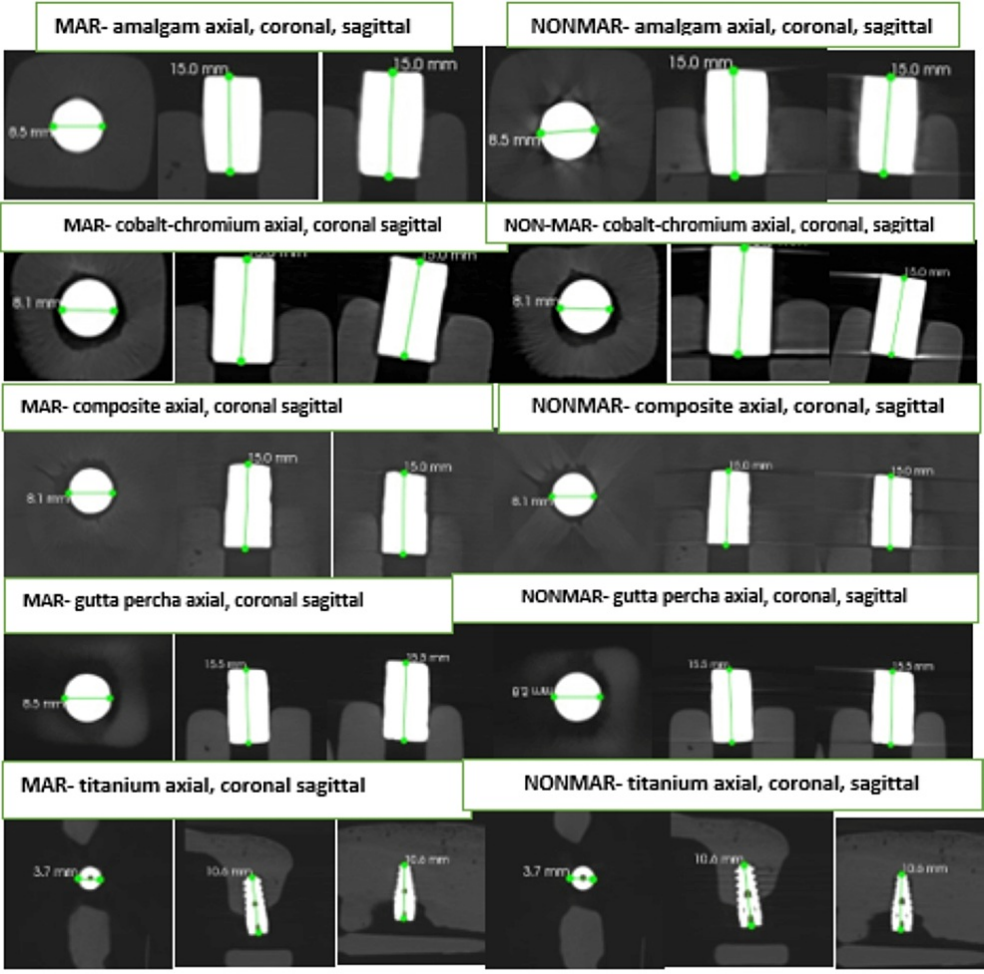
Image showing CS9600 machine of measurement of different metals of length, and width in axial, coronal, sagittal, and artifact surrounding them with and without metal artifact reduction (MAR).

For better accuracy, the artifact was re-checked after an interval of eight days by the same oral and maxillofacial radiologist, and both the tables were matched and submitted for statistical analysis. The Carestream software was used for the measurement of the length and width of the metals which produced the artifact and was evaluated in all three sections (Figure 7).

Image evaluation

In this study, the two oral and maxillofacial radiologists have been blinded to experimental conditions for the measurement of length and width and evaluation of artifacts in CS and ITK-SNAP software. The instruction was received regarding the software tool. Then the examiner evaluated the scan independently by selecting a DICOM file which was uploaded to the software, then the measuring tool was selected for measuring the length and width in all the sections with MAR on and off (Figure 7). This was followed by the evaluation of the artifact according to the numbering system (Table 3).

**Figure 8:**
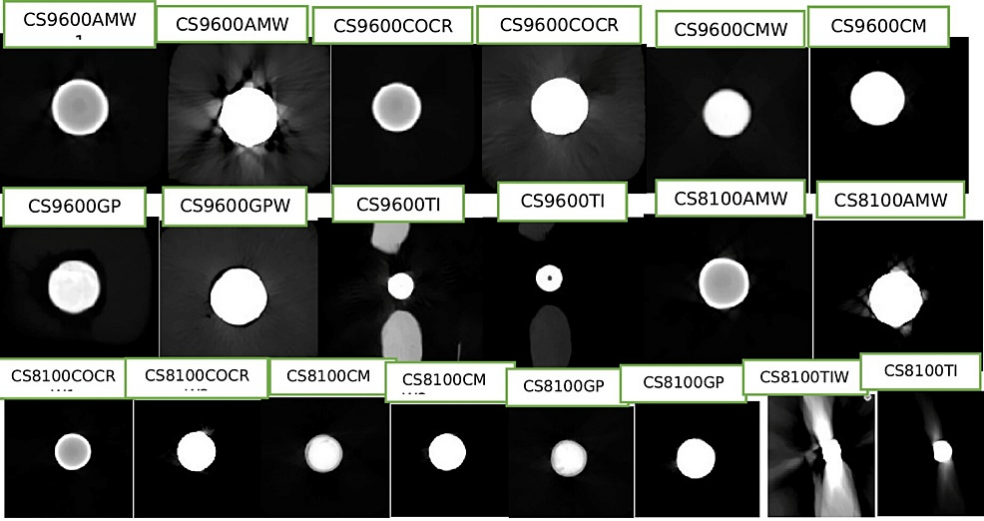
WINDOWING (W1-high window level, large window width W2- Low window level low window width) of different metals in axial, coronal, and sagittal sections.

For windowing, we used ITK-SNAP software which was uploaded as a DICOM file. The contrast tool used for changing the windowing value and the automatic value of W1 and W2 of all high-density material was reduced to a value of 1000 and 3000 respectively (Figure 8). Finally, all data was exported in Excel sheet format (Microsoft, Redmond, WA, USA) as a master chart (Table 4).

**Table 4:** Physical, metal artifact reduction (MAR), and non-MAR measurement of all the metals by using different cone beam computed tomography (CBCT) units.

METALS	AMALGAM	COBALT-CHROMIUM	COMPOSITE	GUTTA-PERCHA	TITANIUM
LENGTH	15.0mm	15.0mm	15.0mm	15.5mm	10.6mm
WIDTH	8.5mm	8.1mm	8.1mm	8.5mm	3.7mm
MAR CS9600	15.0mm	15.0mm	15.0mm	15.5mm	10.6mm
NON-MAR CS9600	15.0mm	15.0mm	15.0mm	15.5mm	10.6mm
MAR CS9600	8.5mm	8.1mm	8.1mm	8.5mm	3.7mm
NON-MAR CS9600	8.5mm	8.1mm	8.1mm	8.5mm	3.7mm
MAR CS8100	15.0mm	15.0mm	15.0mm	15.5mm	10.6mm
NON-MAR CS8100	15.0mm	15.0mm	15.0mm	15.5mm	10.6mm
MAR CS8100	8.5mm	8.1mm	8.1mm	8.5mm	3.7mm
NON-MAR CS8100	8.5mm	8.1mm	8.1mm	8.5mm	3.7mm
MAR ACTEON	15.0mm	15.0mm	15.0mm	15.5mm	10.6mm
NON-MAR ACTEON	15.0mm	15.0mm	15.0mm	15.5mm	10.6mm
MAR ACTEON	8.5mm	8.1mm	8.1mm	8.5mm	3.7mm
NON-MAR ACTEON	8.5mm	8.1mm	8.1mm	8.5mm	3.7mm
NON-MAR CS9300	15.0mm	15.0mm	15.0mm	15.5mm	10.6mm
NON-MAR CS9300	8.5mm	8.1mm	8.1mm	8.5mm	3.7mm
NON-MAR NEWTOM	15.0mm	15.0mm	15.0mm	15.5mm	10.6mm
NON-MAR NEWTOM	8.5mm	8.1mm	8.1mm	8.5mm	3.7mm
NON-MAR DENTIUM	15.5mm	15.5mm	15.5mm	16.0mm	11.0mm
NON-MAR DENTIUM	9.0mm	9.0mm	8.5mm	8.5mm	4.0mm

Statistical analysis

All the data was entered in the Excel sheet and analyzed using SPSS (Statistical Package for Social Sciences) 21.0 version (IBM Corp., Armonk, NY, USA). The data was analyzed for probability distribution using the Kolmogorov-Smirnov test, where the p-value of <0.05 indicated that the data were not normally distributed.

Descriptive statistics were performed, and length and width were compared using the Wilcoxon sign rank test. Comparison of categorical variables was done using the Chi-square test where the p-value of <0.05 was considered statistically significant.

## Results

The difference in the length and width of all the metals samples as measured using MAR and non-MAR CBCT was found to be statistically non-significant (p-value >0.05) (Table 5).

**Table 5:** Comparison of length and width of five metals samples as assessed by metal artifact reduction (MAR) and non-MAR cone beam computed tomography (CBCT). ªWilcoxon sign rank test - P valueª >0.05 statistically non-significant. AM: amalgam, CO-CR: cobalt-chromium, TI: titanium, CM: composite, GP: gutta-percha

		Median AM	Inter-quartile range	Median CO-CR	Inter-quartile range	Median TI	Inter-quartile range	Median CM	Inter-quartile range	Median GP	Inter-quartile range	P valueª >.05 >.05
CBCT length	MAR	15.0	15.0-15.0	15.0	15.0-15.0	10.6	10.6-10.6	15.0	15.0-15.0	15.5	15.5-15.5	
	Non-MAR	15.0	15.0-15.12	15.0	15.0-15.125	10.6	10.6-10.7	15.0	15.0-15.125	15.5	15.5-15.625	
CBCT width	MAR	8.5	8.5-8.5	8.1	8.1-8.1	3.7	3.7-3.7	8.1	8.1-8.1	8.5	8.5-8.5	
	Non-MAR	8.5	8.5-8.62	8.1	8.1-8.325	3.7	3.7-3.7	8.1	8.1-8.2	8.5	8.5-8.5	

**Figure 9:**
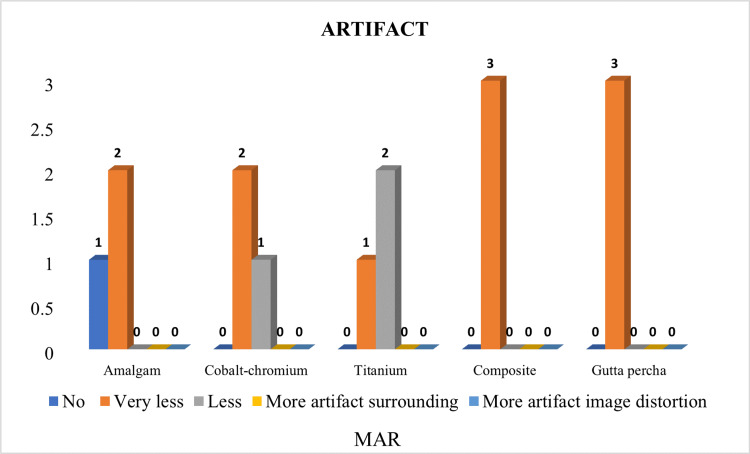
Distribution of samples based on artifacts amongst different materials using metal artifact reduction (MAR) cone beam computed tomography (CBCT).

There was no significant difference in the type of artifact seen with different materials using MAR CBCT (p-value >0.05), which means that MAR effectively reduced metal artifacts produced by high-density restorative materials (Table 6). Distribution of samples based on artifacts amongst different materials using MAR CBCT is shown in Figure 9.

**Table 6:** Comparison of artifacts amongst different materials using metal artifact reduction (MAR) cone beam computed tomography (CBCT). βChi-square test. P valueª >0.05 statistically non-significant.

			GROUP					Total	Chi-square value	df	P value^β^
			Amalgam	Cobalt-Chromium	Titanium	Composite	Gutta Percha				
Artifact	No	Count	1	0	0	0	0	1	10.608	8	.225
		Percentage	33.3%	0.0%	0.0%	0.0%	0.0%	6.7%			
	Very less	Count	2	2	1	3	3	11			
		Percentage	66.7%	66.7%	33.3%	100.0%	100.0%	73.3%			
	Less	Count	0	1	2	0	0	3			
		Percentage	0.0%	33.3%	66.7%	0.0%	0.0%	20.0%			
	More artifact surrounding	Count	0	0	0	0	0	0			
		Percentage	0.0%	0.0%	0.0%	0.0%	0.0%	0.0%			
	More artifact image distortion	Count	0	0	0	0	0	0			
		Percentage	0.0%	0.0%	0.0%	0.0%	0.0%	0.0%			
Total		Count	3	3	3	3	3	15			
		Percentage	100.0%	100.0%	100.0%	100.0%	100.0%	100.0%			

**Figure 10:**
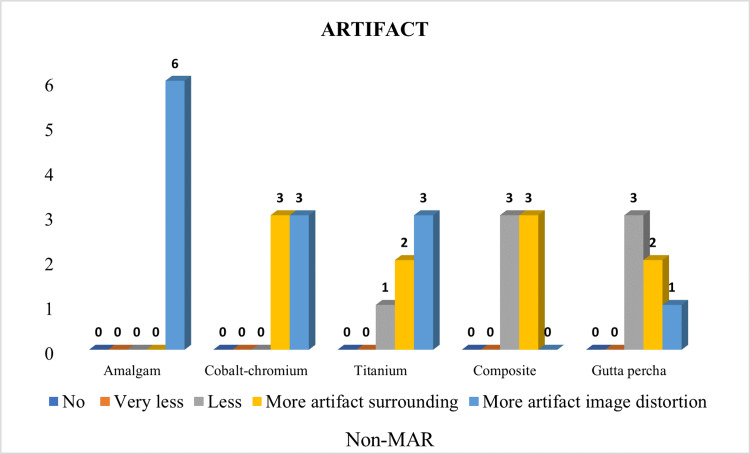
Distribution of samples based on artifacts amongst different materials using non-metal artifact reduction (MAR) cone beam computed tomography (CBCT).

There was a significant difference in the type of artifact seen with different materials on using non-MAR CBCT (p-value <0.05). More artifact image distortion was significantly greater with amalgam and whereas it was significantly less with composite (p-value <0.05) (Table 7). Distribution of samples based on artifacts amongst different materials using non-MAR CBCT is shown in Figure 10.

**Table 7:** Comparison of artifacts amongst different materials on using non-metal artifact reduction (MAR) cone beam computed tomography (CBCT). βChi-square test. *P value <0.05 was considered statistically significant.

			GROUP					Total	Chi-square value	df	P value^β^
			Amalgam	Cobalt-Chromium	Titanium	Composite	Gutta Percha				
Artifact	No	Count	0	0	0	0	0	0	17.725	8	.023*
		Percentage	0.0%	0.0%	0.0%	0.0%	0.0%	0.0%			
	Very less	Count	0	0	0	0	0	0			
		Percentage	0.0%	0.0%	0.0%	0.0%	0.0%	0.0%			
	Less	Count	0	0	1	3	3	7			
		Percentage	0.0%	0.0%	16.7%	50.0%	50.0%	23.3%			
	More artifact surrounding	Count	0	3	2	3	2	10			
		Percentage	0.0%	50.0%	33.3%	50.0%	33.3%	33.3%			
	More artifact image distortion	Count	6	3	3	0	1	13			
		Percentage	100.0%	50.0%	50.0%	0.0%	16.7%	43.3%			
Total		Count	6	6	6	6	6	30			
		Percentage	100.0%	100.0%	100.0%	100.0%	100.0%	100.0%			

## Discussion

Nowadays 3D CBCT imaging is widely used in dentistry, as an important diagnostic tool, and has many advantages as compared to 2-dimensional (2D) imaging [1]. But artifact is one of the major disadvantages which is produced during CBCT scanning [5].

MAR algorithm

To the best of our knowledge, very few studies were carried out on metal artifact reduction algorithms using CBCT, and no evidence of study was found on the use of the MAR algorithm with five to six different CBCT machines.

Bechara et al. 2012 published a study on the reduction of metal artifacts and noise on the MAR algorithm using two CBCT devices, and the authors concluded that MAR was effective in reducing the metal artifact and noise, but MAR increases the reconstruction time of the scan. In our study, similar findings were seen [6]. Panjnoush et al. 2016 implemented a study on the effect of tube current, kilovoltage, metal position, and metal type on the metal artifact in CBCT images and the author found that there was a significant negative influence in CBCT images by metal positions and tube current, and increasing the KVP was found to be beneficial. In our study, we could not change KVP and tube current which was fixed by the manufacturer [4]. Queiroz et al. 2016 conducted a study on different metals including dental amalgam alloy, and gutta percha, and found a positive influence on significant reduction of metal artifact produced by dental amalgam, but gutta percha did not produce enough image artifact to be significantly reduced by MAR. Similarly in our study composite, and gutta percha did not produce enough artifacts to be reduced by the MAR algorithm [6]. Queiroz et al. 2017 evaluated the influence of FOV and voxel size similar to the CBCT algorithm, and they found a negative influence of FOV and voxel size on the CBCT images of dental materials [4,7]. de Faria Vasconcelos et al. 2018 conducted a study on different metals, FOV, and metal positions, which concluded that the artifacts produced by different dental materials and different FOV can be significantly reduced by the MAR algorithm, with no effect on metal position [8]. Queiroz et al. 2018 conducted a study on several basis images and the MAR algorithm was seen to significantly reduced the metal artifact with fewer radiation doses [9]. de Faria Vasconcelos et al. 2020 evaluated the volume correction and influence of material using three CBCT devices considering the metal position and FOV. They found that there was no impact of metal position and FOV using the MAR algorithm, but a significant difference was found in a volume of a cylinder [10].

In our study, data was evaluated irrespective of FOV, metal position, voxel size with respect to physical dimensions and MAR algorithm. We found that different machines had different calibration systems, and out of the six machines, four machines had fixed exposure parameters as set by the manufacturing company, and in the remaining two machines the exposure parameter can be changed according to the FOV and other factors [11]. We scanned all metals with small FOV using different CBCT machines measured. The difference in the length and width of all these five metals using MAR, non-MAR, and digital caliper device measurements was found to be statistically non-significant (p-value >0.05) (Table 5, 6, 7) [12].

Codari et al. 2017 mentioned in their study that amalgam produced strong artifacts, while titanium produced low artifacts. In our study, similar findings were seen with amalgam, and the proportion of images with ‘more artifact image distortion’ was significantly greater in a non-MAR group (p-value <0.05). The proportion of images with ‘very less artifact’ was significantly greater in the MAR group (p-value <0.05) although gutta percha produced artifact surrounding it, no distortion of the image was observed. The proportion of images with ‘less artifact’ and ‘more artifact surrounding’ was significantly greater in the non-MAR group (p-value <0.05) and the proportion of images with ‘very less artifact’ was significantly greater in the MAR group (p-value <0.05) (Figure 9, 10) [13].

Panjnoush et al. 2016 described the artifact produced by the cobalt-chromium as severe as compared to titanium as the atomic number of cobalt and chromium is higher than titanium. In our study, we found similarities in artifacts produced by cobalt-chromium and titanium, with the CO-CR proportion of the images with ‘more artifact surrounding’ and ‘more artifact image distortion’ being significantly greater in a non-MAR group (p-value <0.05) [4].

In the TI proportion of images with ‘very less artifact’ was significantly greater in the MAR group (p-value <0.05). In composite, the proportion of images with ‘less artifact’ and ‘more artifact surrounding’ was significantly greater in a non-MAR group (p-value <0.05). The proportion of images with ‘very less artifact’ was significantly greater in the MAR group (p-value <0.05) [4,13,14] (Figure 9, 10).

Basically, two types of artifacts produced by the metals come under beam hardening, namely cupping artifacts called metal distortion artifacts and extinction artifacts called missing value artifacts [15,16]. During the evaluation it was revealed that MAR did not completely remove the artifact and was not effective for gutta-percha and composite material. Bar diagram indicating the category of ‘very less artifact’ was still present with metals using MAR with no significant difference found [17].

In some CBCT machines, the MAR tool is not available, in such a situation the physician can change the exposure parameters to improve the image quality [18].

Windowing adjustment

Basically, windowing is widely used in the medical field, and very less in the dental field. It is nothing but the gray value or gray tone measurement and change of contrast value [19]. Coelho-Silva et al. 2020 implemented the windowing concept using the ITK-SNAP software. W1 significantly reduced the volumetric distortion when they compared W2 in all conditions [4]. We evaluated W1 and W2 images of AM and COCR showing, W1 induced more volumetric distortion, and reduce the density of metal, with significant artifact reduction seen, and W2 induced significant artifact, distortion of the size of metal, and little volumetric distortion. The remaining metals i.e., CM, and GP, showed little volumetric distortion with W1, and both W1, and W2 showed no effect on an artifact, and TI showed different behavior with a different machine [20].

The Hounsfield unit (HU) number, the grayscale brightness, and the contrast can be altered by changing the HU number. To the best of our knowledge, this software would be beneficial in reducing high-density metal artifacts [21].

Limitations

The major drawback of the present study is the very limited sample size and parameters analyzed. So future studies involving a large number of samples and analysis of the effect of various parameters including kVp, mA, FOV, and windowing on metal artifact reduction are required for making a definitive conclusion.

## Conclusions

No significant difference was found in the length and width of the metals between MAR and non-MAR tools. The high-density restorative material materials including amalgam and cobalt-chromium produced more artifacts which were significantly reduced by MAR, while with titanium the number of images with different degrees of the artifact was not found to differ significantly between MAR and non-MAR groups and gutta-percha produced fewer artifacts which significantly reduced by the MAR.

In windowing, W1 reduced the artifact but reduced the volume and density of metals. W2 produced metal artifacts and increased the density and volume of the metal. Thus, the influence of large window width and high window level would be beneficial to reduce the metal artifact and increase the image quality.
